# Improving genomic predictions by correction of genotypes from genotyping by sequencing in livestock populations

**DOI:** 10.1186/s40104-019-0315-z

**Published:** 2019-01-24

**Authors:** Xiao Wang, Mogens Sandø Lund, Peipei Ma, Luc Janss, Haja N. Kadarmideen, Guosheng Su

**Affiliations:** 1 Center for Quantitative Genetics and Genomics, Department of Molecular Biology and Genetics, Aarhus University, Tjele, Denmark; 2 Department of Bio and Health Informatics and Department of Applied Mathematics and Computer Science, Technical University of Denmark, Kongens Lyngby, Denmark; 3 School of Agriculture and Biology, Shanghai Jiaotong University, Shanghai, China

**Keywords:** Genomic prediction, Genotype correction, Genotyping by sequencing, Simulation

## Abstract

**Background:**

Genotyping by sequencing (GBS) is a robust method to genotype markers. Many factors can influence the genotyping quality. One is that heterozygous genotypes could be wrongly genotyped as homozygotes, dependent on the genotyping depths. In this study, a method correcting this type of genotyping error was demonstrated. The efficiency of this correction method and its effect on genomic prediction were assessed using simulated data of livestock populations.

**Results:**

Chip array (Chip) and four depths of GBS data was simulated. After quality control (call rate ≥ 0.8 and MAF ≥ 0.01), the remaining number of Chip and GBS SNPs were both approximately 7,000, averaged over 10 replicates. GBS genotypes were corrected with the proposed method. The reliability of genomic prediction was calculated using GBS, corrected GBS (GBSc), true genotypes for the GBS loci (GBSr) and Chip data. The results showed that GBSc had higher rates of correct genotype calls and higher correlations with true genotypes than GBS. For genomic prediction, using Chip data resulted in the highest reliability. As the depth increased to 10, the prediction reliabilities using GBS and GBSc data approached those using true GBS data. The reliabilities of genomic prediction using GBSc data were 0.604, 0.672, 0.684 and 0.704 after genomic correction, with the improved values of 0.013, 0.009, 0.006 and 0.001 at depth = 2, 4, 5 and 10, respectively.

**Conclusions:**

The current study showed that a correction method for GBS data increased the genotype accuracies and, consequently, improved genomic predictions. These results suggest that a correction of GBS genotype is necessary, especially for the GBS data with low depths.

**Electronic supplementary material:**

The online version of this article (10.1186/s40104-019-0315-z) contains supplementary material, which is available to authorized users.

## Background

Genotyping by sequencing (GBS) can produce multiplex libraries of samples based on restriction enzyme and DNA barcoded adapters, and potentially reduce the cost of genotyping [1]. With the reduced-representation sequencing of multiplexed samples, GBS has been developed as a robust method to discover and genotype genome-wide molecular markers [2]. For some species, a commercial chip array is not available, thus GBS will be a good approach to obtain genotypes of DNA markers [3]. However, genotyping quality of GBS tends to be lower than for a chip array [4]. Since genome-wide sequence read depth varies along each sequenced genome of different individuals, genotype quality also varies accordingly [5]. Therefore, the proportion of correctly called genotypes will decrease after decreasing read depths.

Several studies have suggested that it is more powerful to sequence more individuals at the lower coverage [6]. Low-coverage sequencing could capture as much of the variation across the genome as SNP arrays and yielded a commensurate increase in statistical power, which would be a more attractive strategy for the studies of complex trait genetics [7, 8]. In Gorjanc’s report [4], expanding the training set resulted in higher overall accuracy of estimated breeding value (EBV), even with reducing the quality of genotyping for lower expense, but genotyping quality may be more important for the prediction set. It was shown that prediction accuracy increased greatly when read depths also increased in the prediction set [4].

Due to the lower coverage, heterozygous genotypes wrongly genotyped as homozygotes are considered to be a serious problem in GBS data. For example, a read depth of one would genotype only one allele of a diploid at random, so that a true *Aa* genotype is definitely genotyped into *aa* or *AA* genotype by mistake. Previous studies have proposed the maximum-likelihood (ML) method for calling genotypes in low-coverage sequencing data [9, 10], and also developed related programs, such as ANGSD [11] and polyRAD [12]. The R package polyRAD estimated a posterior probability from the priors and likelihoods for each individual and allele using Bayes’ theorem. It applied information from high-depth markers to improve genotyping accuracy of low-depth markers using population structure and linkage between loci [12], Additionally, some studies investigated relationship estimation for better relatedness matrices construction using GBS with low depth [13, 14].

In practice, it is possible to correct the wrong genotype calls of GBS data based on read depths and allele frequencies and, consequently, improve the GBS quality to some extent. Therefore, genotype error correction methods are required to complement the future use of GBS data [8]. Simulation is a highly valuable tool to assess such GBS correction methods. Thus, the objective of this study was to propose a method of genotype correction for original GBS data, and then investigate the improvement of genomic prediction (GP) using the simulated data of livestock populations. In this study, four different read depths of GBS genotypes and chip array (Chip) genotypes were simulated. Breeding values were predicted using GBS, corrected GBS (GBSc) and Chip genotypes. The accuracies of genomic predictions were compared to assess the value of GBS and the improvement of GBSc from genotype correction using different genotype data sets.

## Methods

In this study, genomic and phenotypic data of ten replicates for each scenario were generated by QMSim software (version 1.10) [15]. Parameters used for generating the population structure and genome are given in Table 1 and Table 2.

**Table 1:** Simulation parameter of population structure

Step	Population structure	Value
	Number of replicates	10
	Overall heritability	0.3
	QTL heritability	0.3
	Phenotypic variance	1.0
Step1: Historical generation (HG)	Foundation population size of HG	2000
	Number of generation in phase 1	1000
	Population size in the end of phase 1	2000
	Number of generation in phase 2	200
	Population size in the end of phase 2	400
	Number of male in the last HG	200
	Number of female in the last HG	200
	Number of male from HG	40
	Number of female from HG	200
Step 2: Expanded generation (EG)	Number of generation	1
	Litter size	5
	Proportion of male progeny	50%
	Mating design	Random
	Number of male from EG	100
	Number of female from EG	500
Step 3: Recent generation	Number of generation	10
	Litter size	5
	Proportion of male progeny	50%
	Mating design	Random
	Sire replacement	80%
	Dam replacement	40%
	Selection design	EBV

**Table 2:** Simulation parameter of genome

Genome	Value
Number of chromosome	5
Chromosome length	100 Mb
Number of marker loci on one chromosome	1,000,000
Marker position	Evenly
Number of marker alleles in the first HG	2
Marker allele frequencies in the first HG	Random
Number of QTL loci on one chromosome	100
QTL position	Random
Number of QTL allele in the first HG	2
QTL allele frequency in the first HG	Random
QTL allele effect	From gamma distribution with shape 0.4
Maker mutation rate in the historical population	2.5 × 10^−5^
QTL mutation rate in the historical population	2.5 × 10^−5^

### Population structure

**Fig. 1:**
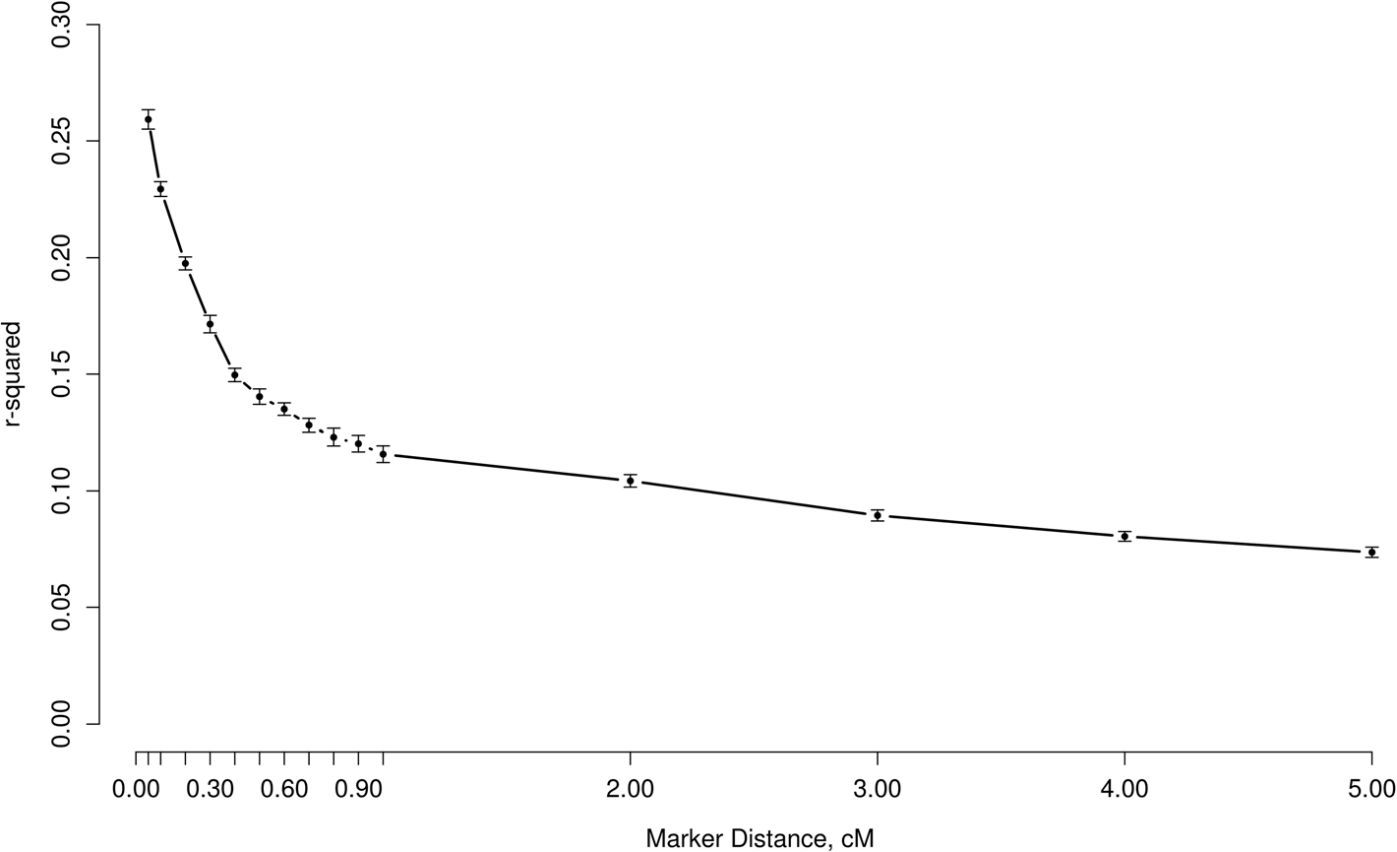
Decay of LD (r-squared) between markers averaged over 10 replicates. Lines combined with solid circle are average r-squared values in the last (10^th^) generations of recent population based on MAF > 0.01 and bars indicate SE

During the historical generations, the foundation population of 2,000 individuals (1,000 males and 1,000 females) was kept at a constant size across 1000 generations, and then reduced gradually to 400 individuals (200 males and 200 females) in the following 200 generations, generating linkage disequilibrium (LD) as a result of the domestication process. Among the 400 individuals in the last generation of historical population, 40 males and 200 females were randomly chosen. Each male mated randomly with five females and each female produced five progeny for population expansion. In the recent generations, 100 males and 500 females from the last generation of the expanded population were selected as the parents of the next generation. This continued for ten generations, keeping a male to mate randomly with five females and each female producing five progeny. Selection and replacement was performed based on EBV. The replacement rate was 80% for males and 40% for females. The breeding values were estimated by best linear unbiased prediction (BLUP) using an animal model [16]. In the whole process of simulation, the individuals of each sex were produced with equal probability based on the random union of gametes, which were sampled from both the male and female gamete pools. The overall heritability, quantitative trait locus (QTL) heritability and phenotypic variance were set as 0.3, 0.3 and 1.0, respectively. No remaining polygenic effect was simulated, so all the genetic variance was explained by QTLs. The phenotypes were created by adding random residuals to the true breeding values (TBV); TBVs were defined as the sum of individual QTL additive effects. The targeted level of LD in this study was close to the values for cattle [17, 18] and pig [19]. The decay of LD between the markers is shown in Fig. 1, which indicates that the mean r-squared of LD in the last (10^th^) generation of the population was 0.259 (SE = 0.004) based on markers with interval less than 50 kb (0 ~ 0.05 cM) and minor allele frequencies (MAF) > 0.01, averaged over 10 replicates.

### Genome

Initial LD was created by the process of mutation-drift equilibrium in the historical generations. In this process, mutation and drift were considered as the only two evolutionary forces due to no selection, no migration and random mating. Crossovers were simulated to be randomly located across the chromosome and the number of crossovers was sampled from a Poisson distribution. A total of 5 × 10^6^ SNP markers were evenly distributed on five chromosomes of length 100 Mb. Allele frequencies of the bi-allelic markers and QTLs were initiated through randomly sampling from a uniform distribution in the first historical generation. In total, 500 QTLs were simulated and randomly distributed on these five chromosomes. Thus, QTL positions for each replicate were different due to the random distribution. QTL allele effects were sampled from gamma distribution with the shape parameter equal to 0.4. The original QTL effect was that one allele had effect and the other allele had zero effect, and then QTL allelic effects at each locus were scaled as (Allelic effect – QTL mean of population) ×$\frac{\sqrt{\mathrm{Defined}\ \mathrm{QTL}\ \mathrm{variance}}}{\sqrt{\mathrm{Observed}\ \mathrm{QTL}\ \mathrm{variance}}}$, where the observed QTL variance were the sum of QTL variances in the last historical generation. By the scaling, the sum of QTL variances in the last historical generation equals to the defined QTL variance (total additive genetic variance in this study). The effect sizes of two alleles of 500 QTLs in one replicate are shown in Additional file 1. In order to establish mutation-drift equilibrium in historical generations, marker and QTL recurrent mutation rates in historical population were both set as 2.5 × 10^− 5^. The recurrent mutations assumed that a mutation altered an allele to another instead of creating a new allele and these transition probabilities were equal. The number of mutations for one chromosome of an individual was sampled from a Poisson distribution with the mean *u* (*u* = 2 × number of loci × mutation rate), and then each mutation was assigned to a random locus in the genome. However, recurrent mutations were generally very rare and there was no evidence that these mutations contributed significantly to the erosion of LD between SNPs [20]. In the recent populations, no mutations were generated.

### Creating GBS and chip array data

**Fig. 2:**
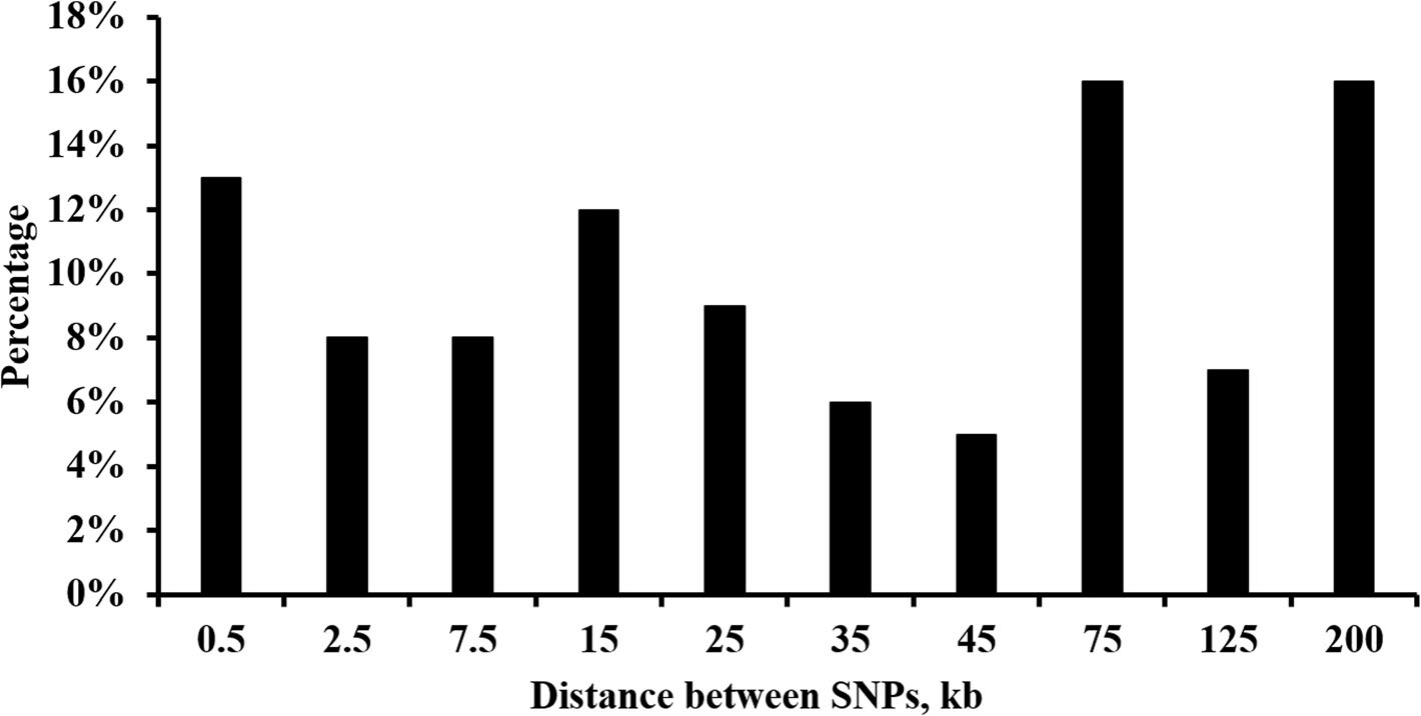
Distribution of distances between the neighboring SNPs

De Donato et al. [21] reported that the distribution of distances between the GBS SNPs differed to that from a chip array data in cattle. For GBS data, the percentage of neighboring SNP less than 50 kb was 44.0% and of more than 150 kb was 13.8%. Following the results of De Donato et al. [21], the distribution of the distances between the neighboring SNPs in this study were set as 13, 8, 8, 12, 9, 6, 5, 16, 7 and 16% for 0.5 kb, 2.5 kb, 7.5 kb, 15 kb, 25 kb, 35 kb, 45 kb, 75 kb, 125 kb and 200 kb, respectively (Fig. 2).

Sequencing errors were not simulated, but low read depths could result in incorrect genotype calls with each allele sampled from Binomial distribution $p\sim B\left(n,\frac{1}{2}\right)$. Thus, the probability of a heterozygous genotype being correctly called was $1-2{\left(\frac{1}{2}\right)}^n$ (e.g., 0.00 when *n* = 1, 0.50 when *n* = 2, and 0.25 when *n* = 3). In other words, the probability of a true heterozygous genotype being wrongly called as one of the observed homozygous genotype was${\left(\frac{1}{2}\right)}^n$. In the simulation, the number of reads (*n*) per locus was drawn from a Poisson distribution *n*~*P*(*x*), where *x* was the average depth (*x* = 2, 4, 5, 10). Actually, the distribution of reads might not reflect the observed distributions accurately due to many factors influencing the variability. Nevertheless, it should not affect the comparisons of genomic prediction results, even though the simulated data has much less variability than observed in practice [22].

We created the GBS genotypes at a heterozygous locus by sampling a random number (*r*) from a uniform distribution *r*~*U*(0, 1). If $r\le {\left(\frac{1}{2}\right)}^n$, the heterozygous genotype was replaced by *aa*. If ${\left(\frac{1}{2}\right)}^n<r<2{\left(\frac{1}{2}\right)}^n$, the heterozygous genotype was replaced by *AA*, else the heterozygous genotype was correctly assigned as *Aa*. Afterwards, the GBS genotypes *aa*, *Aa* and *AA* were recorded as 0, 1 and 2, respectively.

Quality control criteria of call rate ≥ 0.8 and MAF ≥ 0.01 for SNPs were used to edit the GBS data. After quality control, missing genotypes (zero reads) were set as the mean genotype value for the same loci before genomic prediction. In addition, chip array (Chip) data with no sequencing errors or missing genotypes was also simulated for comparison. The SNP markers of Chip were evenly distributed on five chromosomes with a distance of 50 kb between two adjacent markers.

### Genotype correction for GBS data

The genotype correction in this study is to adjust GBS genotypes according to Bayes’ conditional probability *P*(*G*| *GBS*), where *G* is the true genotype (unknown) and *GBS* is the GBS genotype (known) which is subject to genotyping errors. The expected genotype dosage after genotype correction can be rounded to GBSc genotype types (most probable GBSc genotype, i.e., dosage < 0.5: *aa*; dosage > 1.5: *AA*; else: *Aa*). Right and false correction of GBSc were displayed when comparing GBSc genotypes with GBS and true (GBSr) genotypes (Table 3). Homozygous genotypes of GBSc could be corrected into heterozygous genotypes or keep the same homozygous genotypes. If the homozygosity was corrected into heterozygosity and the true genotype of GBSr was also heterozygous, this kind of genotype correction was right genotype correction. However, such genotype correction could be false genotype correction when the true genotype of GBSr was homozygous.

**Table 3:** Genotype changes of corrected GBS (GBSc) in the lower panel of Fig. 3

Genotype change in the lower panel of Fig. 3	GBS	GBSc	GBSr
Right correction of GBSc (GBS ≠ GBSc = GBSr)	aa/AA	Aa	Aa
False correction of GBSc (GBSc ≠ GBS = GBSr)	aa/AA	Aa	aa/AA

If *GBS*_*aa*_ (genotype is labeled in the subscript for the nomenclature) is observed, there are two possible true genotypes (*G*_*aa*_ and *G*_*Aa*_), and the probabilities are

$$ P\left({G}_{aa}|{GBS}_{aa}\right)=\frac{P\left({G}_{aa}\right)\ P\left({GBS}_{aa}|{G}_{aa}\right)\ }{P\left({GBS}_{aa}\right)}, $$

$$ P\left({G}_{Aa}|{GBS}_{aa}\right)=1-P\left({G}_{aa}|{GBS}_{aa}\right). $$

Similarly, If *GBS*_*AA*_ is observed,

$$ P\left({G}_{AA}|{GBS}_{AA}\right)=\frac{P\left({G}_{AA}\right)\ P\left({GBS}_{AA}|{G}_{AA}\right)\ }{P\left({GBS}_{AA}\right)}, $$

$$ P\left({G}_{Aa}|{GBS}_{AA}\right)=1-P\left({G}_{AA}|{GBS}_{AA}\right). $$

If *GBS*_*Aa*_ is observed, *G*_*Aa*_ is the only possible true genotype,

$$ P\left({G}_{Aa}|{GBS}_{Aa}\right)=1. $$

Let assume *p* = *P*(*A*) and *q* = *P*(*a*) for the true genotype, the relevant probabilities can be written as:

$$ P\left({GBS}_{aa}\right)=P\left({G}_{aa}\right)+P\left({GBS}_{aa}|{G}_{Aa}\right)={q}^2+2 pq{\left(\raisebox{1ex}{$1$}\!\left/ \!\raisebox{-1ex}{$2$}\right.\right)}^n, $$

$$ P\left({G}_{aa}|{GBS}_{aa}\right)=P\left({G}_{aa}\right)\ast P\left({GBS}_{aa}|{G}_{aa}\right)/P\left({GBS}_{aa}\right)={q}^2/P\left({GBS}_{aa}\right), $$

$$ P\left({G}_{Aa}|{GBS}_{aa}\right)=P\left({G}_{Aa}\right)\ast P\left({GBS}_{aa}|{G}_{Aa}\right)/P\left({GBS}_{aa}\right)=2 pq{\left(\raisebox{1ex}{$1$}\!\left/ \!\raisebox{-1ex}{$2$}\right.\right)}^n/P\left({GBS}_{aa}\right), $$

$$ P\left({GBS}_{AA}\right)=P\left({G}_{AA}\right)+P\left({GBS}_{AA}|{G}_{Aa}\right)={p}^2+2 pq{\left(\raisebox{1ex}{$1$}\!\left/ \!\raisebox{-1ex}{$2$}\right.\right)}^n, $$

$$ P\left({G}_{AA}|{GBS}_{AA}\right)=P\left({G}_{AA}\right)\ast P\left({GBS}_{AA}|{G}_{AA}\right)/P\left({GBS}_{AA}\right)={p}^2/P\left({GBS}_{AA}\right), $$

$$ P\left({G}_{Aa}|{GBS}_{AA}\right)=P\left({G}_{Aa}\right)\ast P\left({GBS}_{AA}|{G}_{Aa}\right)/P\left({GBS}_{AA}\right)=2 pq{\left(\raisebox{1ex}{$1$}\!\left/ \!\raisebox{-1ex}{$2$}\right.\right)}^n/P\left({GBS}_{AA}\right). $$

Let 0, 1, and 2 denote *aa*, *Aa* and *AA* genotype, respectively. The original GBS genotypes are scored as *GBS*_*aa*_ = 0, *GBS*_*Aa*_ = 1 and *GBS*_*AA*_ = 2. The correction of GBS used in this study is to correct GBS homozygous genotypes to be expected genotype dosage. Thus,

$$ {GBSc}_{aa}=2 pq{\left(\raisebox{1ex}{$1$}\!\left/ \!\raisebox{-1ex}{$2$}\right.\right)}^n/\left({q}^2+2 pq{\left(\raisebox{1ex}{$1$}\!\left/ \!\raisebox{-1ex}{$2$}\right.\right)}^n\right), $$

$$ {GBSc}_{AA}=\left(2{p}^2+2 pq{\left(\raisebox{1ex}{$1$}\!\left/ \!\raisebox{-1ex}{$2$}\right.\right)}^n\right)/\left({p}^2+2 pq{\left(\raisebox{1ex}{$1$}\!\left/ \!\raisebox{-1ex}{$2$}\right.\right)}^n\right), $$

$$ {GBSc}_{Aa}=1. $$

Allele frequency can be calculated from the data including all reads when assuming Hardy-Weinberg equilibrium. It can be estimated from the known GBS data in this way:

$$ P\left({GBS}_{AA}\right)-P\left({GBS}_{aa}\right)=\left({p}^2+2 pq{\left(\raisebox{1ex}{$1$}\!\left/ \!\raisebox{-1ex}{$2$}\right.\right)}^n\right)-\left({q}^2+2 pq{\left(\raisebox{1ex}{$1$}\!\left/ \!\raisebox{-1ex}{$2$}\right.\right)}^n\right)={p}^2-{q}^2=2p-1, $$

$$ p=\frac{P\left({GBS}_{AA}\right)-P\left({GBS}_{aa}\right)+1\ }{2} $$

### Statistical analysis

Based on the GBS, GBSc, GBSr and Chip data, genomic breeding values (GEBV) were predicted by a SNP-BLUP model using the BayZ package (http://www.bayz.biz/). The model was

$$ \mathbf{y}=\mathbf{1}\upmu +\mathbf{Mg}+\mathbf{e}, $$

where **y** was the vector of phenotypic values, **1** is the vector of ones, μ is the overall mean, **g** is the vector of random unknown marker effects to be estimated, **M** is the coefficient matrix of genotypes which links **g** to **y**, and **e** is the vector of random residuals. It was assumed that $\mathbf{g}\sim N\ \left(0,\mathbf{I}{\sigma}_g^2\right)$.

### Validation

In the 6^th^ to 9^th^ generations of the recent population, 7,500 individuals were used as a training set, in which all individuals were genotyped and phenotyped. The test set comprised of 2,500 genotyped individuals from the 10th generation. The reliabilities of genomic predictions using marker data from GBS, GBSc, GBSr and Chip were compared. The reliabilities of genomic predictions were calculated as squared correlations between the predicted and true breeding values for individuals in the test data set.

## Results

### Distributions of read depth (***n***) at four levels of mean read depth (***x***)

Additional file 2 showed the realized frequency distributions of reads at four mean depths (*x* = 2, 4, 5, 10), which were highly consistent with the theoretical frequencies of Poisson distribution. The percentages of read depth ≤ 5 at mean read depth = 2, 4, 5 and 10 were 98.3%, 78.5%, 61.6% and 6.71%, respectively. The percentages of missing genotypes at mean read depth = 2, 4, 5 and 10 were 13.5%, 1.83%, 0.673% and 0.00464%, respectively. Standard deviation (SD) of ten replicates were all less than 4.74 × 10^− 5^.

### Incorrect genotype calls

It was expected that a heterozygous genotype may be wrongly assigned to a homozygous genotype with probability of $2{\left(\frac{1}{2}\right)}^n$. The upper panel of Additional file 3 shows the proportions of incorrect genotype calls over true genotypes and the SDs of ten replicates were all less than 6.32 × 10^− 3^. Although the average number of realized reads for different loci was nearly the same, there was much variation in the proportion of incorrect genotypes observed for different loci (lower panel of Additional file 3). Having a large proportion of incorrect genotypes in the loci close to QTL regions could affect genomic prediction.

### Improvement of accuracies of GBS genotype by genotype correction

**Fig. 3:**
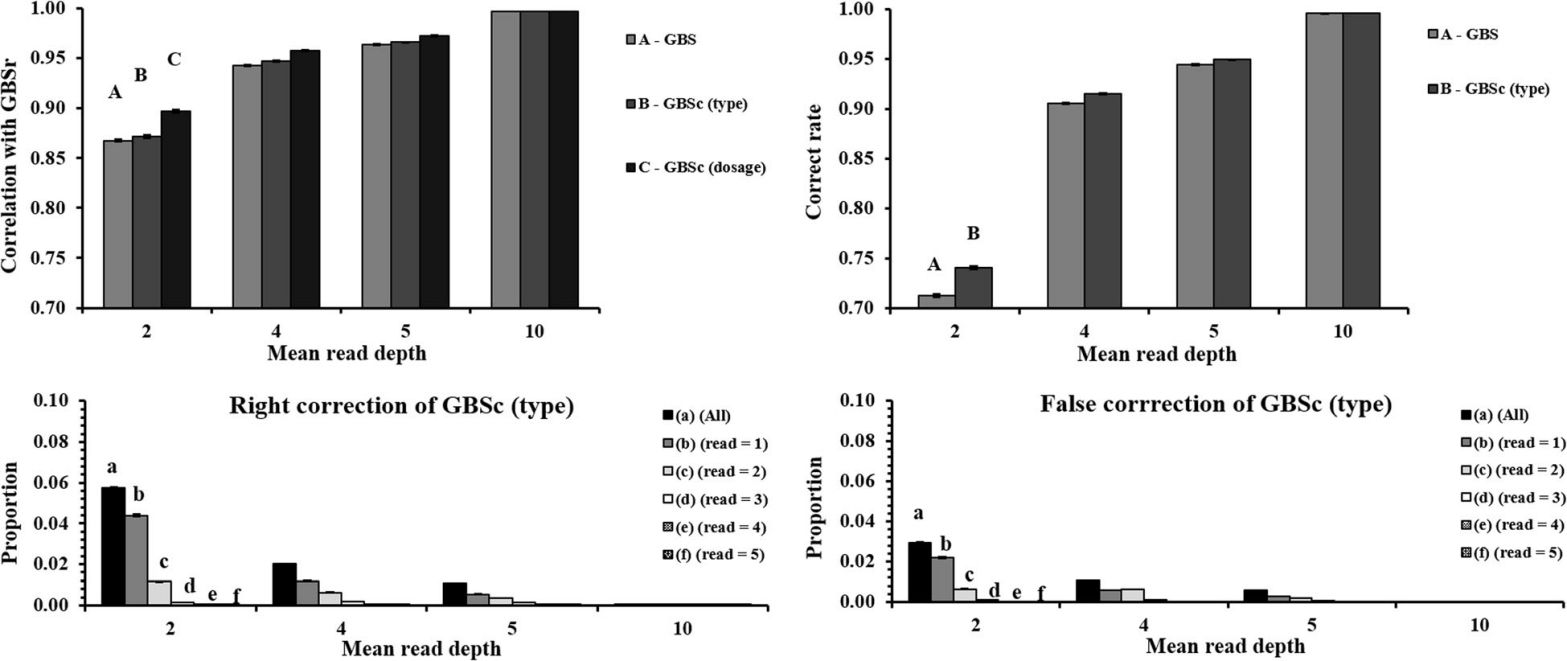
Correlations and correct rates for original GBS (GBS), corrected GBS genotype type (GBSc type), corrected GBS genotype dosage (GBSc dosage) with true genotype in GBS loci (GBSr) data (the upper panel), as well right and false genotype correction of GBSc (type) data (the lower panel) at four mean depths, averaged over 10 replicates

The accuracies of GBS genotypes were measured as correlations between the true genotype and the GBS genotype, as well correct rates of GBS genotype calls. As shown in the upper panel of Fig. 3, genotype correction improved the accuracies of GBS genotype. The correlations between reported genotype and true genotype were highest for the GBSc genotype dosage and lowest for the original GBS, while GBSc genotype type (most probable genotype) was in between. The differences among these three genotype data were larger for lower depth, and not for depth = 10. Similarly, correct rates of GBSc genotype type were higher than those of original GBS for depth = 2, 4, and 5, but not for depth = 10. GBSc genotype type occupied a larger proportion of right genotype correction than false genotype correction (lower panel of Fig. 3). As expected, larger improvement was observed in the genotype data with lower depth.

### Reliabilities of genomic prediction

After quality control (call rate ≥ 0.8 for loci and MAF ≥ 0.01), the number of GBS SNPs obtained for the four depths was approximately 7,000 averaged over 10 replicates. As shown in the Additional file 2, the missing genotypes at mean depth = 2, 4, 5 and 10 were less than 13.5%, so call rate criteria of 80% for loci had nearly no effect on genotypes editing. However, the missing genotypes at mean depth = 1 were high, up to approximately 30%; therefore, a large number of loci did not meet this criterion, and this depth was discarded. In addition, approximately 7,000 SNPs remained for the Chip data using the same quality control criteria (call rate ≥ 0.8 for loci and MAF ≥ 0.01).

**Fig. 4:**
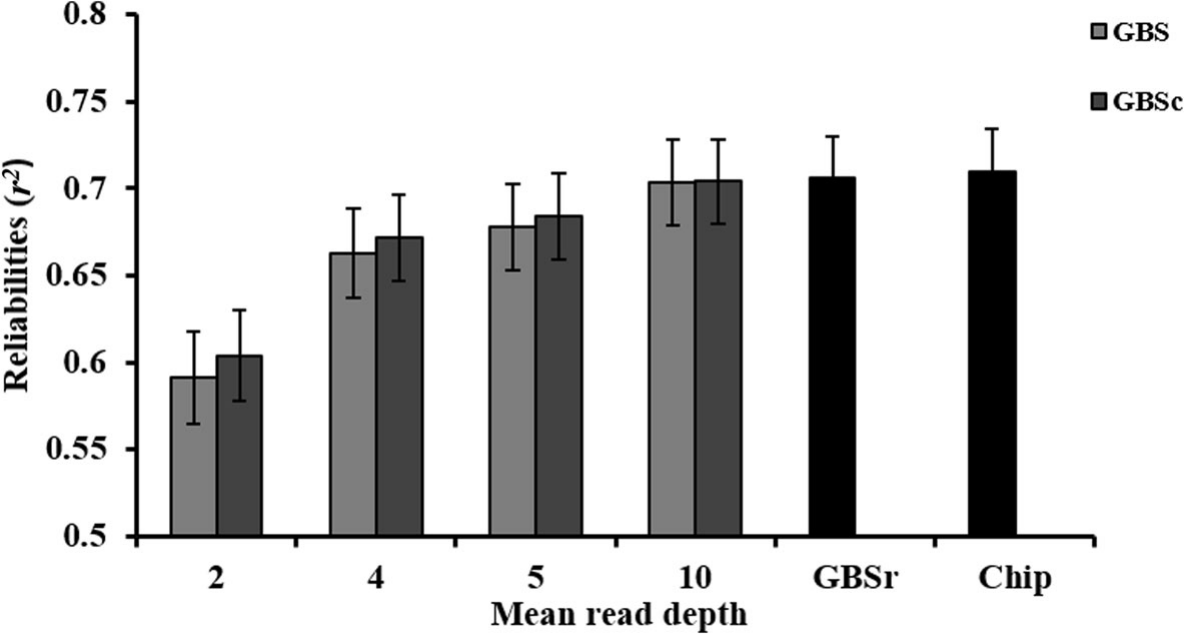
Reliabilities (*r*^2^) of genomic prediction using original GBS (GBS), corrected GBS (GBSc dosage), true genotype in GBS loci (GBSr) and chip array (Chip) data, at four depths, averaged over 10 replicates. Bars indicate SE

The reliabilities (*r*^2^) of genomic prediction averaged over 10 replicates at four depths are shown in Fig. 4. Prediction reliability using Chip data (0.710) was slightly higher than that using true genotype for the GBS loci (GBSr) (0.706). As depth increased from 2 to 10, the prediction reliabilities using GBS and GBSc data gradually approached the reliabilities using GBSr data. The worst prediction reliability was from depth = 2 due to having the most missing and incorrect genotypes (Additional file 2 and Additional file 3). Genotype correction improved genomic prediction to different degrees, consistent with the accuracies of corrected genotypes (Fig. 3). Thus, the reliabilities of genomic prediction using GBSc data were higher than those using GBS data at all four depths (Fig. 4). The standard error (SE) of prediction reliabilities in 10 replicates was approximately 0.025 for scenarios of different depths and types of the marker data.

Without quality control (call rate ≥ 0.8 for loci and MAF ≥ 0.01) for editing genotype data, approximately 8,000 GBS SNPs were obtained at the four depths, averaged over 10 replicates. Table 4 reveals that reliabilities of genomic prediction using GBS and GBSc data before editing genotypes were better than those using the data after editing genotypes. In addition, GBSc led to higher reliability than GBS no matter that correction was performed before or after genotype editing.

**Table 4:** Reliabilities of genomic prediction using original GBS (GBS) and corrected GBS (GBSc) data before and after editing genotypes, at four mean depths, averaged over 10 replicates

Reliability (SE)	GBS (after editing genotypes)	GBS (no editing genotypes)	GBSc (after editing genotypes)	GBSc (no editing genotypes)
Depth = 2	0.591 (0.026)	0.598 (0.023)	0.603 (0.026)	0.610 (0.024)
Depth = 4	0.662 (0.025)	0.663 (0.024)	0.671 (0.025)	0.672 (0.024)
Depth = 5	0.678 (0.025)	0.683 (0.023)	0.684 (0.025)	0.687 (0.023)
Depth = 10	0.703 (0.024)	0.704 (0.024)	0.704 (0.024)	0.704 (0.024)

## Discussion

### Potential application of GBS in breeding programs

As a highly multiplexed technology for constructing reduced representation libraries, GBS generates large numbers of SNPs for use in genetic analyses [2]. Unlike genotyping approach of chip arrays, GBS allows de novo marker discovery, even when there is no reference genome [3]. The GBS technology includes digestion by a restriction enzyme, ligation of barcode adapter, amplification by PCR and sequencing of amplified DNA [3]. Repetitive regions of genome can be avoided and lower copy regions can be targeted efficiently to simplify the alignment problems by using appropriate restriction enzymes [2, 23]. Single-well digestion and barcode adapter ligation results in reduced sample handling, less PCR amplification and no size fractionation limitation [24]. Currently, GBS has been widely used in many breeding programs. Poland et al. [25] presented a GBS approach for barley and wheat lacking of a reference genome sequence and found GBS to have broad applications in plant breeding programs. The applications of genomic selection in aquaculture species has been underpinned by GBS techniques, which are available for a handful of aquaculture species [26]. Additionally, GBS has great potential application in domestic species whose reference sequences are either being developed or have been fully sequenced, as it enables acceptable marker density for genomic selection in cattle at one third of the cost of the current genotyping technologies [21].

In this study, the highest reliability of genomic prediction was from using the chip array (Chip) data. Obviously, Chip SNPs were evenly distributed along the genome, so at least one SNP was in strong LD with QTLs. However, large distances between some neighboring GBS SNPs weakened the LD between SNP and QTL. De Donato et al. [21] reported that the BovineSNP50 chip array had a large proportion of intervals from 20 kb to 100 kb and only 3% had an interval larger than 150 kb, while GBS data showed that about 14% of SNP intervals were more than 150 kb. Even if LD exists at long distances (longer than 1 cM in some regions and some species), LD will decay as the distance between marker and QTL increases [27]. This study restricted the numbers of Chip SNPs and GBS SNPs to be same. If the number of GBS SNPs increases, GBS data could perform as well as Chip data in genomic prediction. In general, GBS can produce enough information even at relatively low coverage, but wrong calls of genotype are the main disadvantage that requires further improvement.

### Genotype dosage and most probable genotype

**Fig. 5:**
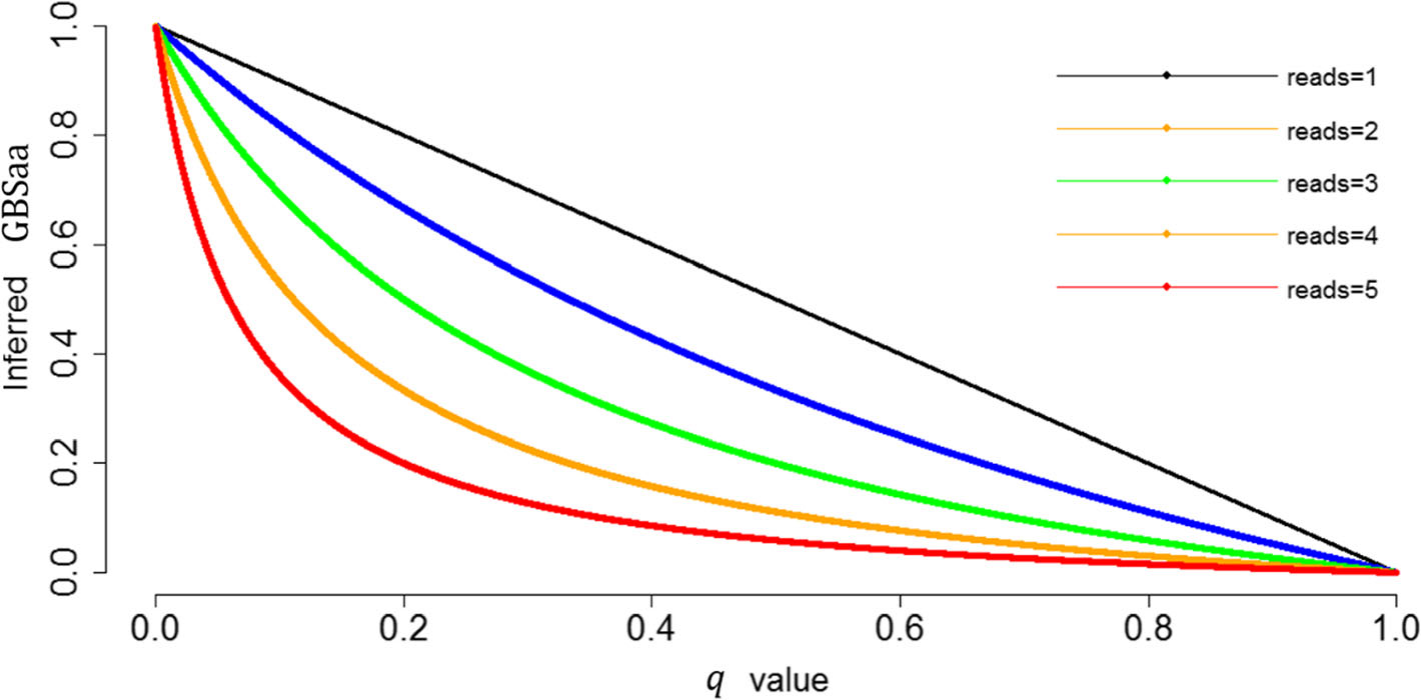
Inferred genotype dosage of *GBS*_*aa*_ versus *q* value for different read depths (*n*)

This study demonstrated the method of correction for GBS genotype to improve the accuracy of GBS data, following the previous studies [9–14]. This correction method resulted in GBSc genotype type and GBSc genotype dosage. The GBSc genotype type was derived from rounding the GBSc genotype dosage. The inferred GBSc genotype dosage of genotype *aa* was $\mathrm{dosage}\left({GBSc}_{aa}\right)=1-\frac{q^2\ }{q^2+2 pq{\left(\frac{1}{2}\right)}^n}=\frac{1}{\frac{q}{2p{\left(\frac{1}{2}\right)}^n}+1}$, which ranges between 0 and 1. For example, dosage(*GBSc*_*aa*_) = 1 − *q* if read depth = 1, which is always larger than 0.5 when *q* is less than 0.5. When reads depths become higher, dosage(*GBSc*_*aa*_) was closer to zero (Fig. 5) and the inferred GBSc genotype dosage and inferred GBSc genotype type were more consistent. The results also indicate that the rounded genotypes derived from dosage were more accurate than original GBS genotype, but some of them might be far from the true genotype, dependent on the number of reads and allele frequency.

### Correction of GBS genotype improved genomic prediction

The proportions of right genotype correction were more than the proportions of false genotype correction in this study, so the correct rates of GBSc increased 0.028, 0.010, 0.005 and 0.001 from original GBS at depth = 2, 4, 5 and 10, respectively (Fig. 3). Therefore, genotype correction increased the accuracy of GBS genotypes. GBSc genotype dosage had higher correlation than GBSc genotype type, so GBSc genotype dosage was more accurate (Fig. 3). In fact, all meaningful information about uncertainty is lost by choosing the largest probability [28], such as the GBSc genotype type rounded from GBSc genotype dosage in this study. For genotype imputation, a general approach is also the use of posterior probabilities. Imputed genotypes are predictions instead of actual observations of genotyping, so incorporating the uncertainty of these predictions could avoid spurious results in some cases [29].

We only used a SNP-BLUP model for predicting breeding value in this study. The result indicated that genomic prediction using corrected GBS data were more accurate than using the original GBS data (Fig. 4). It is expected that the gain in reliability of genomic prediction from genotype correction will also present when using other genomic prediction models such as Bayesian variable selection models, because genotype correction increases the accuracies of genotype assignment (Fig. 3).

In this study, retaining more SNPs resulted in higher prediction reliabilities (Table 4), which meant that a decreasing or less stringent thresholds of call rate and MAF led to an increase in prediction reliability [30]. Our study also revealed that genotype correction improved genomic prediction more than removing the MAF threshold. Cooke et al. [31] expected to reduce genotype errors in GBS data by estimating allelic dropout. Their simulation studies using their GBStools package improved genotyping accuracy more than hard filters [31]. Meanwhile, Furuta et al. [32] used their post SNP-calling error correction to eliminate most errors of raw GBS data, but some remained. Their studies also found that simple imputation methods can reinforce the usefulness of GBS data tremendously, even if up to 75% of missing data for each marker existed in the raw GBS data [32]. However, methods rely on population types and reference genomes, so they may not always be applied.

## Conclusions

The current study demonstrated a method for the correction of GBS genotypes. The results showed that the correction increased the accuracy of GBS genotype and increased the accuracy of genomic prediction. Therefore, a correction method for GBS genotype is necessary, especially for GBS data with low depth.

## Additional files

Additional file 1: Effect sizes of 500 QTLs in one replicate. (TIF 257 kb)

Additional file 2: Read depths at four mean read depths in one replicate. (TIF 171 kb)

Additional file 3: Proportions of wrongly called genotypes at four depths averaged over the whole genome and over 10 replicates (the upper panel), as well proportions of wrongly called genotypes (the lower panel) along 50 loci in one replicate. (TIF 187 kb)

## Abbreviation

BLUP: Best linear unbiased prediction
Chip: Chip array
cM: Centimorgan
EBV: Estimated breeding value
EG: Expanded generation
GBS: Genotyping by sequencing
GBSc: Corrected GBS
GBSr: True genotype in the GBS loci
GEBV: Genomic estimated breeding value
GP: Genomic prediction
HG: Historical generation
LD: Linkage disequilibrium
MAF: Minor allele frequency
Mb: Megabase pair
ML: Maximum likelihood
QTL: Quantitative trait loci
SD: Standard deviation
SE: Standard error
SNP: Single nucleotide polymorphism
TBV: True breeding value
