# Supplementary figures and images for: Improving genomic predictions by correction of genotypes from genotyping by sequencing in livestock populations
Source: J Anim Sci Biotechnol. 2019 Jan 24;10:8. doi: 10.1186/s40104-019-0315-z (PMC6350319; doi:10.1186/s40104-019-0315-z)

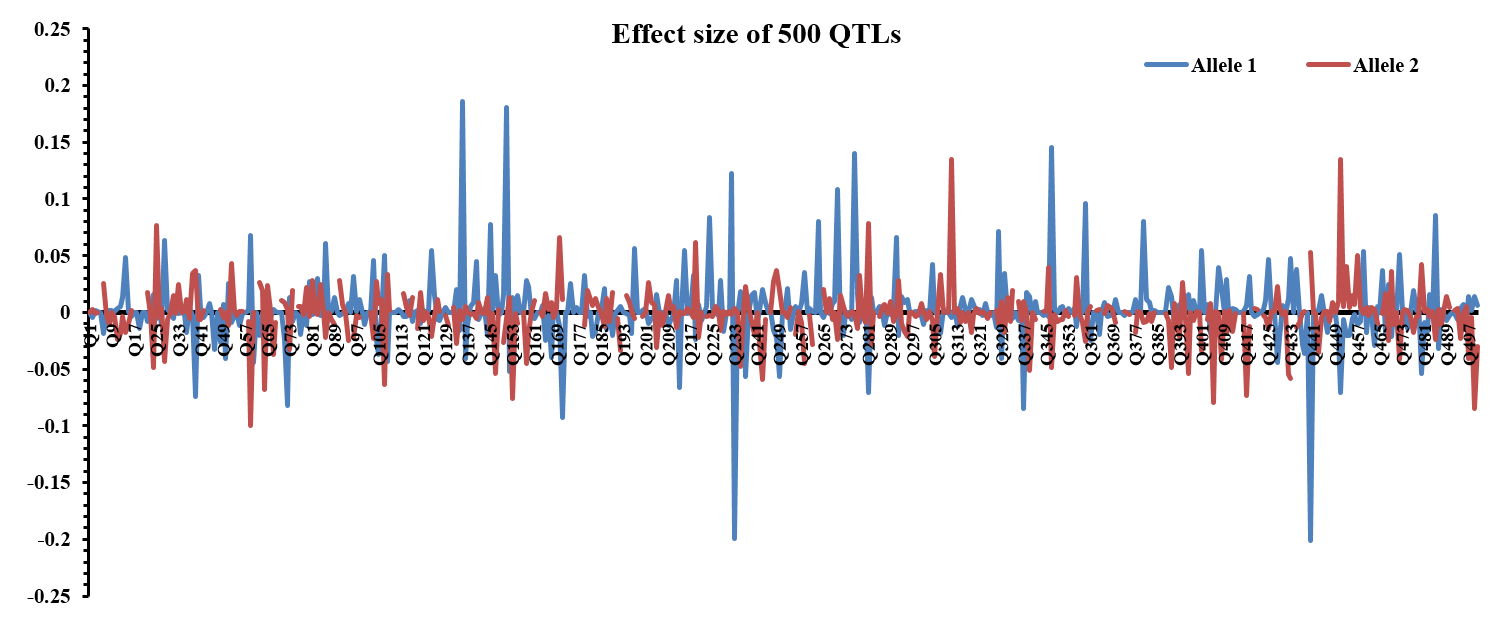

Supplement: Supplementary file 1 — Effect sizes of 500 QTLs in one replicate. (TIF 257 kb) [file 40104_2019_315_MOESM1_ESM.tif]

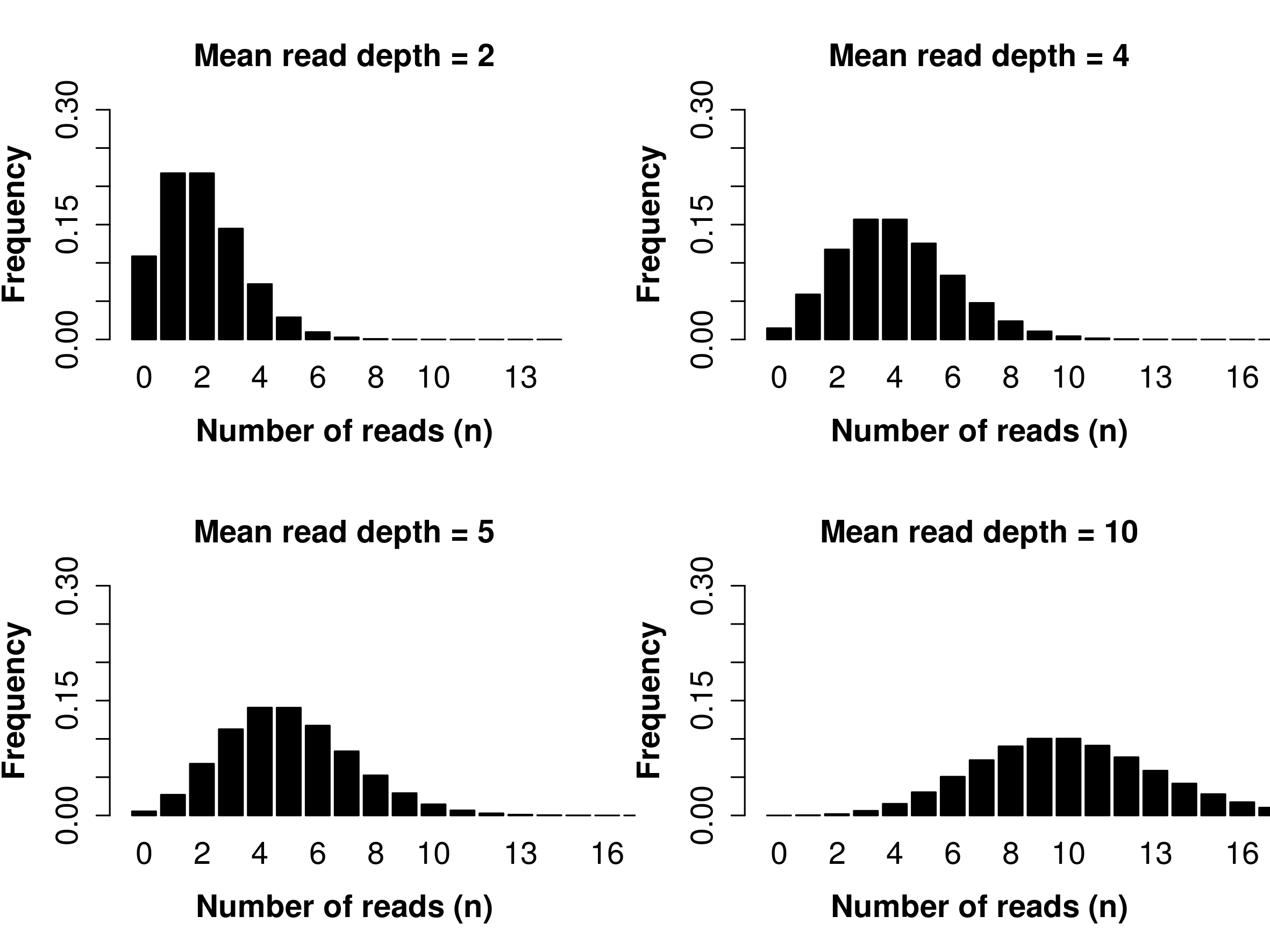

Supplement: Supplementary file 2 — Read depths at four mean read depths in one replicate. (TIF 171 kb) [file 40104_2019_315_MOESM2_ESM.tif]

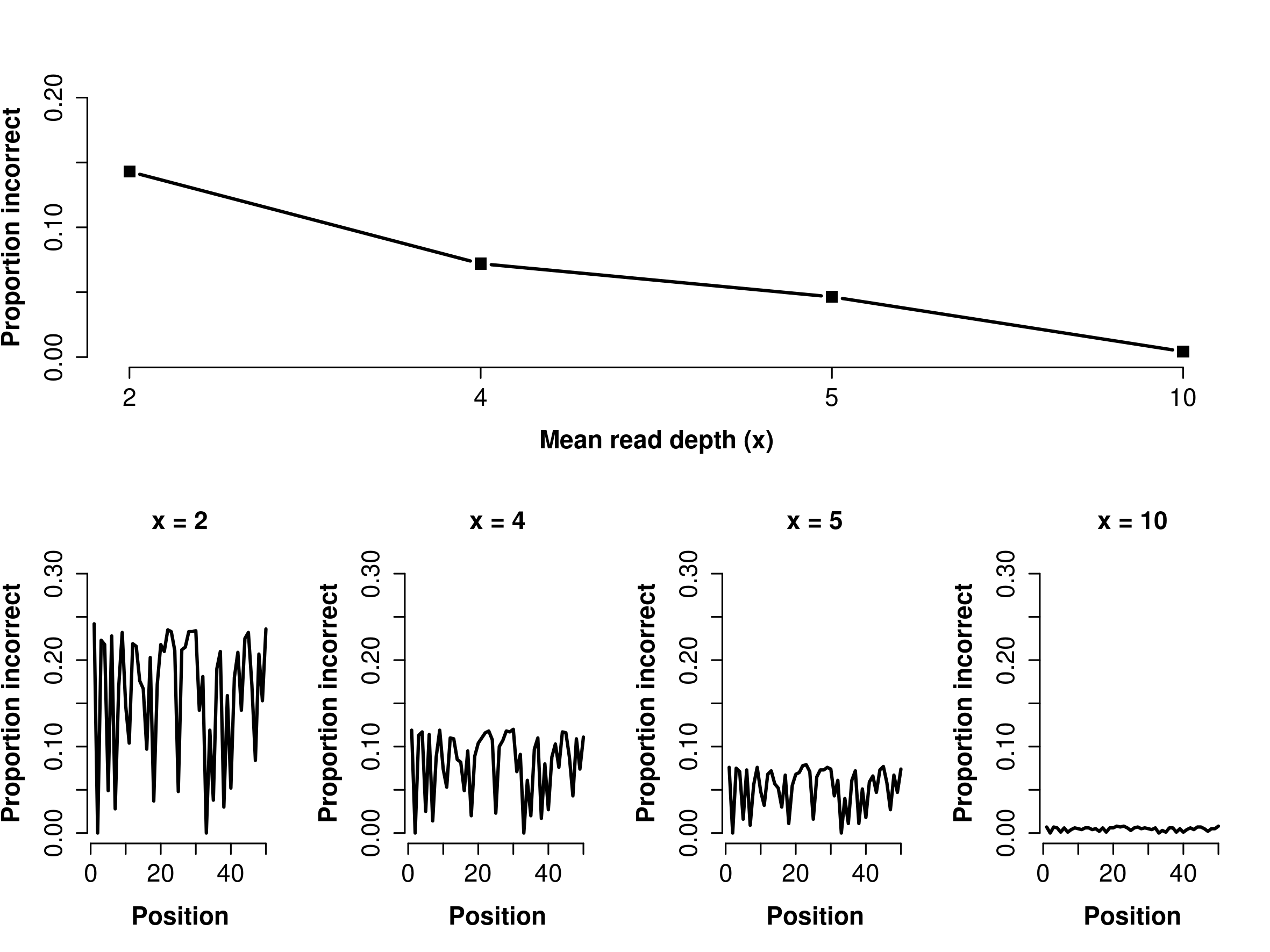

Supplement: Supplementary file 3 — Proportions of wrongly called genotypes at four depths averaged over the whole genome and over 10 replicates (the upper panel), as well proportions of wrongly called genotypes (the lower panel) along 50 loci in one replicate. (TIF 187 kb) [file 40104_2019_315_MOESM3_ESM.tif]
